# The loss of P2X7 receptor expression leads to increase intestinal glucose transit and hepatic steatosis

**DOI:** 10.1038/s41598-017-13300-8

**Published:** 2017-10-10

**Authors:** Guillaume Arguin, Jean-François Bourzac, Morgane Placet, Caroline M. Molle, Michel Paquette, Jean-François Beaudoin, Jacques A. Rousseau, Roger Lecomte, Mélanie Plourde, Fernand-Pierre Gendron

**Affiliations:** 1 Department of Anatomy and Cell Biology, Pavillon de Recherche Appliquée sur le Cancer, Faculty of Medicine and Health Sciences, Université de Sherbrooke, Sherbrooke, QC Canada; 2 Sherbrooke Molecular Imaging Centre of CRCHUS and Department of Nuclear Medicine and Radiobiology, Université de Sherbrooke, Sherbrooke, QC Canada; 3 Department of Medicine, Research Center on Aging, Université de Sherbrooke, Sherbrooke, QC Canada

## Abstract

In intestinal epithelial cells (IEC), it was reported that the activation of the P2X7 receptor leads to the internalization of the glucose transporter GLUT2, which is accompanied by a reduction of IEC capacity to transport glucose. In this study, we used *P2rx7* ^−/−^ mice to decipher P2X7 functions in intestinal glucose transport and to evaluate the impacts on metabolism. Immunohistochemistry analyses revealed the presence of GLUT2 at the apical domain of *P2rx7* ^−/−^ jejunum enterocytes. Positron emission tomography and biodistribution studies demonstrated that glucose was more efficiently delivered to the circulation of knockout animals. These findings correlated with increase blood glucose, insulin, triglycerides and cholesterol levels. In fact, *P2rx7* ^−/−^ mice had increased serum triglyceride and cholesterol levels and displayed glucose intolerance and resistance to insulin. Finally, *P2rx7* ^−/−^ mice developed a hepatic steatosis characterized by a reduction of *Acaca*, *Acacb*, *Fasn* and *Acox1* mRNA expression, as well as for ACC and FAS protein expression. Our study suggests that P2X7 could play a central role in metabolic diseases.

## Introduction

Adenosine 5′-triphosphate (ATP) is secreted in the extracellular environment where it acts as a signaling molecule^1^. Extracellular ATP activates a number of receptors amongst which the ionotropic P2X7 receptor that belongs to the P2X receptor family of gated-ion channel^2^. P2X7 expression is ubiquitous, although, it was often associated with hematopoietic cells where its activity is key for the maturation of IL-1β amongst other functions^3^. In the intestine, P2X7 expression was found in intestinal epithelial cells (IEC)^4–7^, as well as in neurons of the enteric nervous system where it regulated intestinal motility^8^ and post-infection visceral pain^9^. Its expression in the intestinal *lamina propria* of patients suffering from Crohn’s disease was associated with increased inflammation^10^. In Peyer’s patches of the mouse ileum, P2X7 stimulated apoptosis of T helper cells in order to maintain appropriate immune responses toward invading bacteria^11^. Aside from its obvious role in immunology, P2X7 was identified as an important regulatory element of metabolism^12^. In fact, *P2rx7* knockout mice under diverse genetic background suffer from hyperglycemia, glucose intolerance, and impaired beta cells function in response to a high-sucrose diet^13^, as well as abnormal fat distribution^14^. It was also reported that absence of P2X7 expression protected *P2rx7* knockout mice from streptozotocin-induced diabetes^15^. The protective effect could be recapitulated by intraperitoneal injection**s** of P2X7 antagonist Brilliant Blue G. It was proposed that P2X7 participated to the development of type 1 diabetes by contributing to the recruitment of antigen presenting cells and lymphocytes to the pancreas and pancreatic lymph node^15^. More recently, P2X7 was identified as an attractive pharmacological target to treat early phase diabetic retinopathy, a common consequence of long-term diabetes^16^. Aside from exacerbated immune responses as reported in both of these studies, diabetes and diabetic retinopathy share a common denominator that is high blood glucose levels and/or poor glycemic regulatory mechanism in which the P2X7 is involved as depicted above. In this context, it was reported that P2X7 expression was upregulated in IEC in response to high glucose concentration^17^. These findings are further supporting the idea that P2X7 expression could be an important receptor in maintaining metabolism homeostasis.

In IEC, the sodium/glucose cotransporter SGLT-1 ensures glucose entries and the subsequent export toward the bloodstream is mediated by the glucose transporter type 2 GLUT2^18^. However, other studies suggested that apical GLUT2 might be required to accommodate the important concentration of luminal glucose in postprandial conditions^19–21^. Despite this debate about the presence and function of GLUT2 at the IEC apical compartment, GLUT2 is required for glucose export to the blood stream, and as such represent an interesting target to control glycaemia. In this context, we previously showed that the stimulation of the P2X7 receptor initiated the internalization of GLUT2 in IEC. This delocalization of GLUT2 was accompanied by a significant reduction of glucose transport by IEC^4^.

In this work, we have used live positron emission tomography (PET) imaging and biodistribution analyses to monitor the enteral absorption and transport of a [^18^F]-FDG/glucose solution in *P2rx7* ^+/+^ and *P2rx7* ^−/−^ mice. Supporting our previous *in vitro* studies^4^, we found that in *P2rx7* ^−/−^ mice the intestine more efficiently transport glucose thus leading to a rapid increase in blood glucose concentrations. Hence, we determined that knockout animals were suffering from symptoms mimicking human metabolic diseases, including hepatic steatosis.

## Results

### GLUT2 jejunum localization in IEC

**Figure 1:**
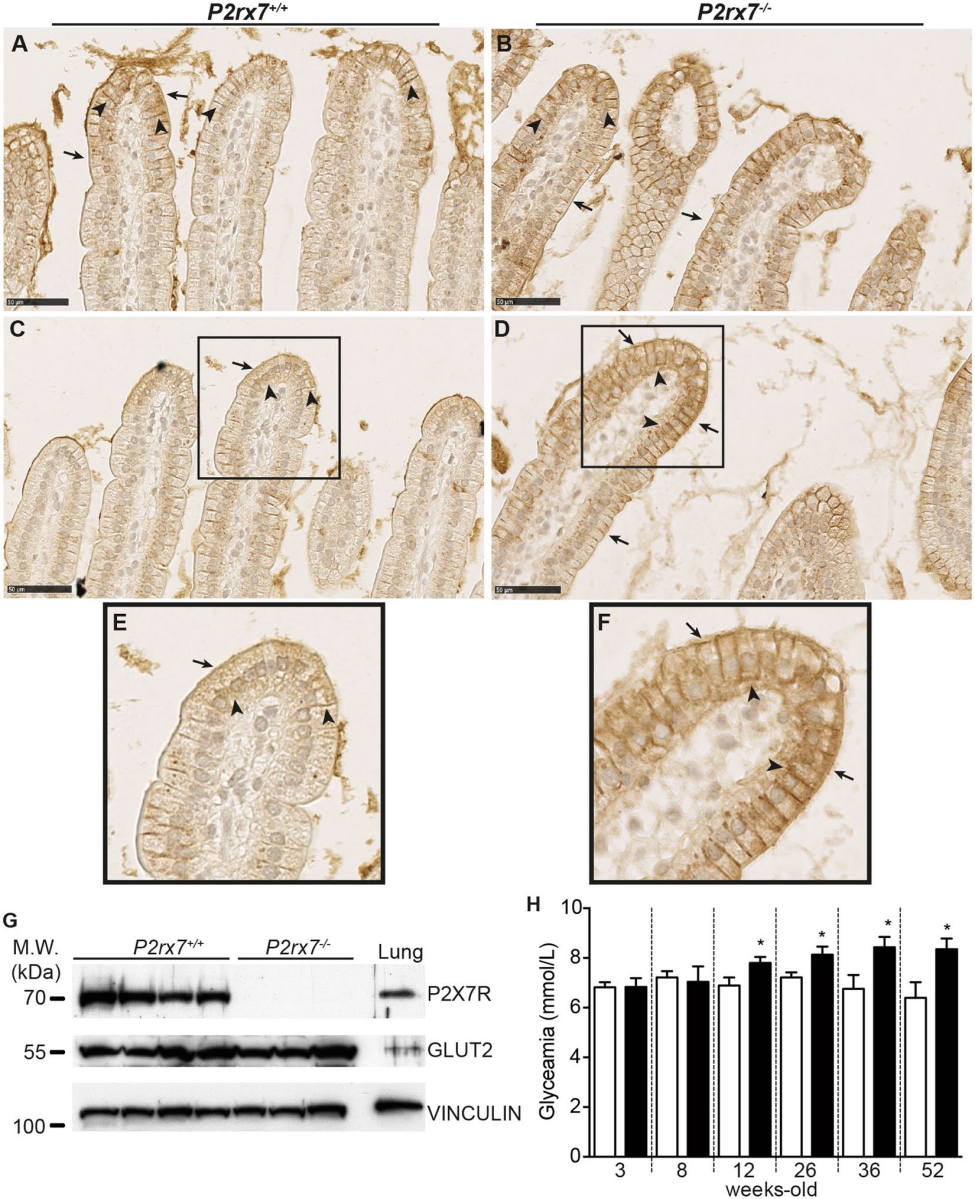
The absence of P2X7 expression modulates GLUT2 localization at the apical compartment of mouse jejunum enterocytes and increases glycaemia. (**A–F**) Immunohistochemical localization of GLUT2 in the jejunum enterocytes of *P2rx7* ^+/+^ (**A**,**C**,**E**) and *P2rx7* ^−/−^ mice (**B**,**D**,**F**). In mouse jejunum, GLUT2 was found mainly located at the apex of villi in both *P2rx7* ^+/+^ and *P2rx7* ^−/−^ mice. We observed a differential localization of GLUT2 with increase apical (black arrows) and basolateral (black arrowheads) signals for GLUT2 in *P2rx7* ^−/−^ mice (**B**,**D**) as compared to *P2rx7* ^+/+^ animals (**A**,**C**). Panels E and F are a 2.5x magnification of the selected area (black box) showed in panels C and D, respectively. The micrographs are representative of four *P2rx7* ^+/+^ mice and four knockout animals with two different animals shown on panels A–D. For panels A to D the scale bars = 50 μm at an original magnification of 40x. (**G**) Western blot analysis of isolated mouse jejunum IEC showing the absence of P2X7 expression in knockout animals and the similar expression of GLUT2 in both animal groups. Whole lung protein lysate was used as a positive control for P2X7 expression and VINCULIN as a control for protein loading and integrity. H) Blood glucose levels were determined after 6 h of diurnal fasting and reported as the mean ± SEM. *P2rx7* ^+/+^ (open bars) and *P2rx7* ^−/−^ (black bars) male mice of different age (n = 5 to 9 animals per groups) were used and the statistical significance determined by an unpaired *t* test, where *p < *0*.*05*.

GLUT2 is constitutively expressed at the basolateral membrane of enterocytes whereas its apical expression is regulated, although the presence of GLUT2 in this compartment is still a matter of debate^18,22,23^. *In vitro*, we determined that P2X7 stimulation induced GLUT2 internalization^4^. To circumvent the potential influence of luminal glucose and insulin on GLUT2 localization^24^, we starved *P2rx7* ^−/−^ mice and wild-type littermates for 6 h prior to experiments. Under these conditions, immunohistochemistry studies showed that GLUT2 was mainly located at the apex of jejunum villosities (Fig. 1A–D). We clearly see the localization of GLUT2 at the enterocyte basolateral membranes (Fig. 1A–F, black arrowheads). GLUT2 staining is barely visible at the apical membrane of *P2rx7* ^+/+^ enterocytes (Fig. 1E, black arrows). In *P2rx7* ^−/−^ mice (Fig. 1F), strong GLUT2 staining was found at the apical compartments (black arrows) and basolateral membranes (black arrowheads) of enterocytes. The apparent modulation of GLUT2 localization at the apical membrane of *P2rx7* ^−/−^ enterocytes cannot be attributable to an increase protein expression since the overall GLUT2 expression in isolated IEC remains the same (Fig. 1G). The presence of apical GLUT2 was accompanied by a significant augmentation in the concentration of blood glucose measured in 12-week-old knockout mice as compared to wild-type littermates (Fig. 1H). The higher glycaemia values were maintained through time. The experiments were stopped at 52 weeks of age, as some mice had serious health issues that included loss of sight, severe obesity and mobility difficulties. In light of these results, we hypothesis that the relocalization of GLUT2 at the apical compartment of *P2rx7* ^−/−^ enterocytes could lead to a more efficient capacity of *P2rx7* ^−/−^ mice to absorb and transport luminal glucose.

### *In vivo* glucose absorption and biodistribution

**Figure 2:**
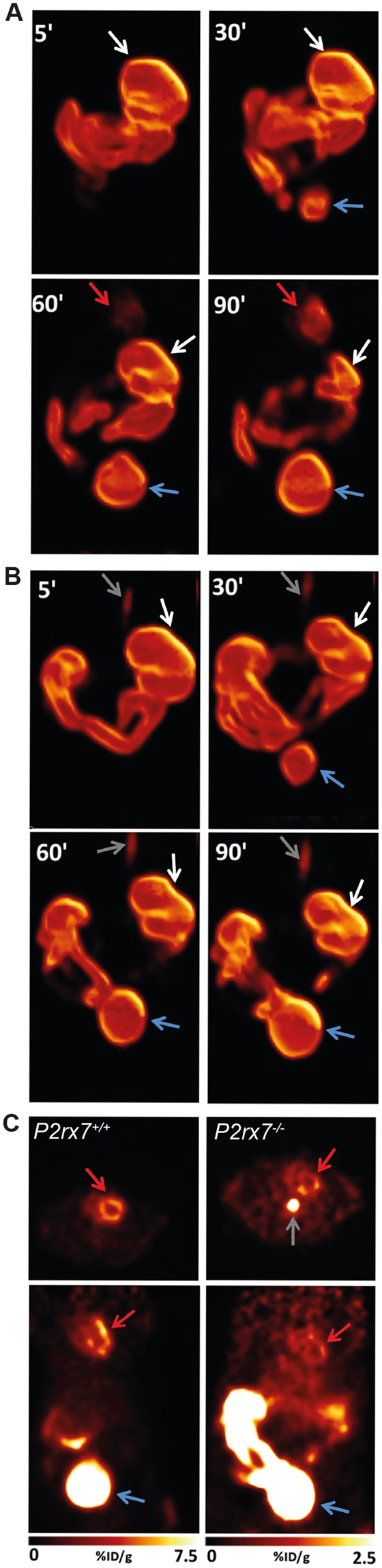
Maximum intensity projection 3D rendering at different time points following *per os* inoculation of FDG in the stomach of (**A**) *P2rx7* ^+/+^ and (**B**) *P2rx7* ^−/−^ mice, showing the progression of FDG through the digestive tract. (**C**) Representative transaxial (*top panel*) and coronal (*bottom panel*) 5-mm thick slices through the heart at 90 minutes post-inoculation showing normal myocardial FDG uptake in a *P2rx7* ^+/+^ mouse (*left*) and significantly reduced uptake in a *P2rx7* ^−/−^ mouse (*right*). Note the 3-fold enhanced image contrast in the *P2rx7* ^−/−^ panel to allow visualization of the low FDG uptake in the heart. The presence of FDG activity in the esophagus results from accidental contamination during the radiotracer administration. White arrows: stomach; Red arrows: heart; Blue arrows: bladder; Gray arrows: esophagus.

**Figure 3:**
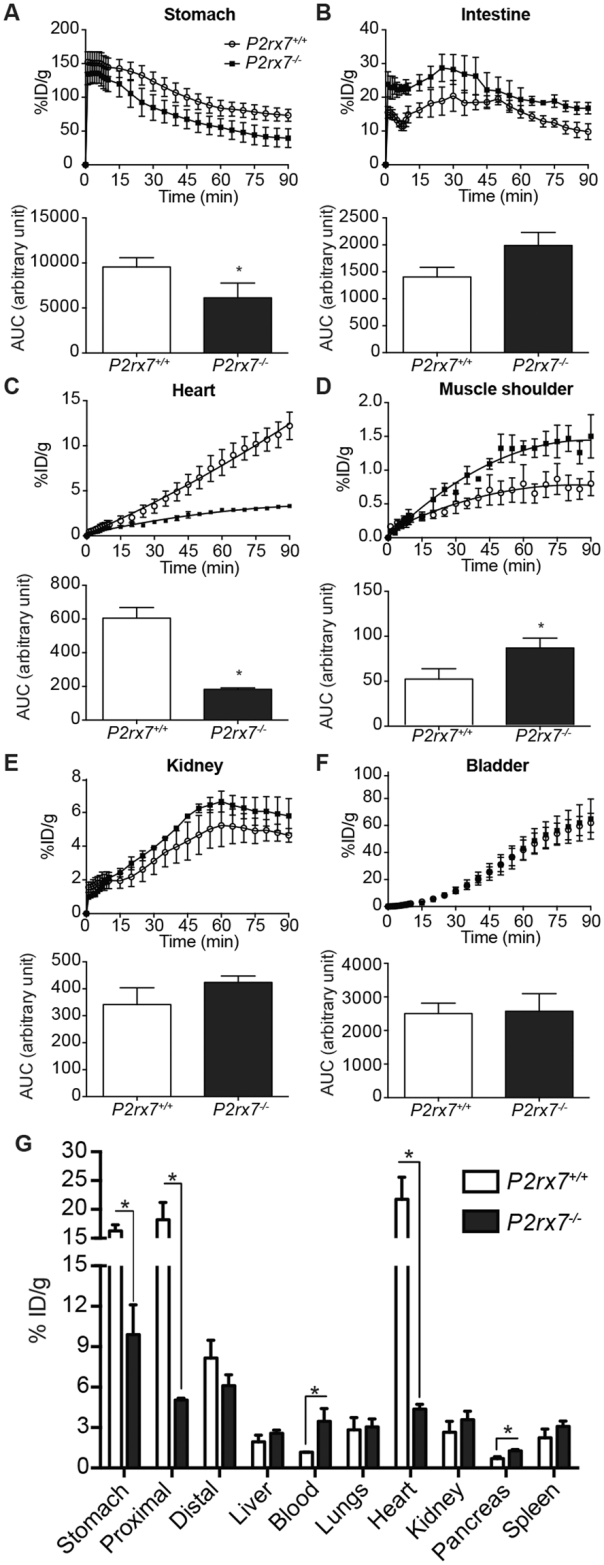
Glucose/[^18^F]-FDG solution is absorbed and distributed at different rates by organs in *P2rx7* ^−/−^ mice. (**A–F**) A dose of 50 mg D-glucose supplemented by [^18^F]-FDG was given *per os* to 6 h-fasted mice and absorption followed live using PET imaging. The upper panels are showing the absorption through time in units of percent injected dose per g of tissue (%ID/g) for the selected organs, whereas the lower panels present the area under the curve (AUC) showing the amount of radiotracer found in the selected organs after 90 min. Results are expressed as the mean ± SEM of the %ID/g for 3 *P2rx7* ^+/+^ and 3 *P2rx7* ^−/−^ mice. Statistical significance was determined by an unpaired *t* test, where *p < 0.05. (**G**) Following the 90 min PET acquisition, the selected organs were harvested and the [^18^F]-FDG uptake measured in a Packard Cobra II E5003 gamma counter. Results are expressed as the mean ± SEM of the %ID/g for 3 *P2rx7* ^+/+^ and 3 *P2rx7* ^−/−^ mice. Statistical significance was determined by an unpaired *t* test, where *p < *0*.*05*.

To study the potential differential efficiency to absorb, transport and distribute glucose, *P2rx7* ^+/+^ and *P2rx7* ^−/−^ mice were gavage with a solution of glucose supplemented with [^18^F]-FDG. The progression of the [^18^F]-FDG tracer through the digestive tract and distribution to other organs was monitored by live PET scans as showed in the movie (video 1.mp4, supplemental data). As we observed in this video, the distribution and progression of the [^18^F]-FDG tracer appears to progress more rapidly through the gastro-intestinal tract of *P2rx7* ^−/−^ mouse when compared to the *P2rx7* ^+/+^ animal. Hence, we noticed that the proximal region of the small intestine of the *P2rx7* ^−/−^ mouse is practically devoid of [^18^F]-FDG signals at the end of the animation, being mostly concentrated in the most distal section of the intestine, when compared to the *P2rx7* ^+/+^ animal. These observations are better illustrated in Fig. 2, where time-frame captions are showed for *P2rx7* ^+/+^ (Fig. 2A) and *P2rx7* ^−/−^ (Fig. 2B) mice. As observed in the animations, the [^18^F]-FDG signal was initially more intense in the intestine of the *P2rx7* ^−/−^ mouse (Fig. 2B) from 5 to 30 min following the gavage when compared to the *P2rx7* ^+/+^ animal (Fig. 2A). At 60 and 90 min, we clearly saw a reduction of the signal intensity in the proximal and mid-intestine and a significant accumulation in the distal intestine of the *P2rx7* ^−/−^ mouse when compared to the lower signal intensity and spread distribution of the tracer in the *P2rx7* ^+/+^ animal. For the *P2rx7* ^+/+^ mouse, we could also see [^18^F]-FDG activity appearing in the heart at 60 and 90 min; a signal absent in the *P2rx7* ^−/−^ animal. This was further illustrated in Fig. 2C, which showed a significantly reduced uptake of [^18^F]-FDG in the heart of *P2rx7* ^−/−^ mouse. This surprising result may be related to the metabolic status of the *P2rx7* ^−/−^ mice as we discussed below. To further support the live imaging, the progression and signal intensity of the [^18^F]-FDG tracer was calculated at different time points in the different organs of interest (Fig. 3).

The transition rate of the [^18^F]-FDG/glucose solution through the stomach is similar between the two groups of mice as illustrated by the two parallel curves of %ID/g of tissue over time (Fig. 3A, upper panel). However, the decreased AUC values (Fig. 3A, lower panel) showed that the retention of glucose in the stomach of *P2rx7* ^−/−^ mouse was significantly less when compared to *P2rx7* ^+/+^ animals. As a consequence, there is a greater amount of the [^18^F]-FDG/glucose solution reaching the intestine of *P2rx7* ^−/−^ mice. This is confirmed by the distribution curves of Fig. 3B, which suggest an increase overall availability of the [^18^F]-FDG/glucose solution in the intestine of *P2rx7* ^−/−^ mouse. This increase activity in *P2rx7* ^−/−^ animals seems inconsistent with the significant and robust reduction in the [^18^F]-FDG quantity found at 90 min in the proximal small intestine of *P2rx7* ^−/−^ mice as compared to *wild-type* animals (Fig. 3G). However, PET images suggest a faster transit of the [^18^F]-FDG through the proximal small intestine of the *P2rx7* ^−/−^ mice, which would be consistent with a lower intestinal uptake of the radiotracer. In addition, it is technically impossible by PET imaging to make a distinction between the [^18^F]-FDG activity found in the intestine lumen vs. the activity localized in the intestinal layers. This limitation explains our inability to measure uptake differences for each of the time points presented in Fig. 3B. Finally, the apparent discrepancies between the observations showed in the video and Fig. 2 and those presented in Fig. 3B could also be attributable to the fact the presented curves are the results of measurements of the [^18^F]-FDG activity collected from the whole small intestine and not from a particular region such as the jejunum. Next, we determine the distribution of [^18^F]-FDG over time in the heart (Fig. 3C) and the vascularized shoulder muscle (Fig. 3D) of mice. For the heart, the accumulation of the [^18^F]-FDG tracer followed a linear steady-state kinetic in both *P2rx7* ^+/+^ and *P2rx7* ^−/−^ mice (Fig. 3C, upper panel) as previously reported^25^. Overall, there is a significant reduction of [^18^F]-FDG signals in the organ of knockout mice, which correlates with a marked reduction of AUC values (Fig. 3C, lower panel) in these animals. The marked reduction in the %ID/g of tissue in the heart of *P2rx7* ^−/−^ mice could be partially explained by the fact that about 70% of the ATP produced in the heart comes principally from fatty acid (FA) oxidation and not from glucose^26^. Hence, the use of FA as the heart energy source is exacerbated in different diabetes rodent models^26^. Since the determination of [^18^F]-FDG uptake by the heart does not seem to adequately reflect the level of circulating glucose, we measured, on the opposite, a significant accumulation of [^18^F]-FDG over time in the vascularized shoulder muscle (Fig. 3D). These latest findings suggest that glucose was more efficiently transported in the circulation of *P2rx7* ^−/−^ than in *P2rx7* ^+*\*+^ mice (Fig. 3D). No significant difference was observed between animal groups for the kidneys (Fig. 3E) and bladder (Fig. 3F).

Following the PET procedures, organs of interest were harvested and [^18^F]-FDG biodistribution measured using a Packard Cobra II E5003 gamma radiation counter. As for the PET studies, a significant reduction in the level of [^18^F]-FDG was measured in the stomach and heart of *P2rx7* ^−/−^ mice as compared to wild-type animals (Fig. 3G). As stated above, contrary to the PET measurement for which the intestine was measured as a whole, the biodistribution of [^18^F]-FDG was markedly and significantly reduced in the proximal region of the small intestine (duodenum and proximal jejunum) of *P2rx7* ^−/−^ mice as compared to *P2rx7* ^+/+^ animals (Fig. 3G). It is worth mentioning that the proximal small intestine is the region where most of the glucose is absorbed under normal conditions^23^. No significant difference was observed for the distal small intestine (distal jejunum and ileum). The reduction in the level of [^18^F]-FDG in the proximal small intestine of knockout animals and increase in blood, suggest that glucose is rapidly absorbed by enterocytes and transported to the blood stream, thus supporting the idea that the P2X7 receptor is involved in the regulation of glucose entries as we previously showed *in vitro* ^4^. A slight, but significant increase in [^18^F]-FDG concentration was also measured in the pancreas of *P2rx7* ^−/−^ for which the [^18^F]-FDG levels increase from 0.69 ± 0.14 %ID/g of tissue for *wild-type* mice to 1.27 ± 0.10 %ID/g in *P2rx7* ^−/−^ animals (Fig. 3G).

### *P2rx7* deficient mice display higher glycaemia, increase weight gain and dyslipidemia

**Figure 4:**
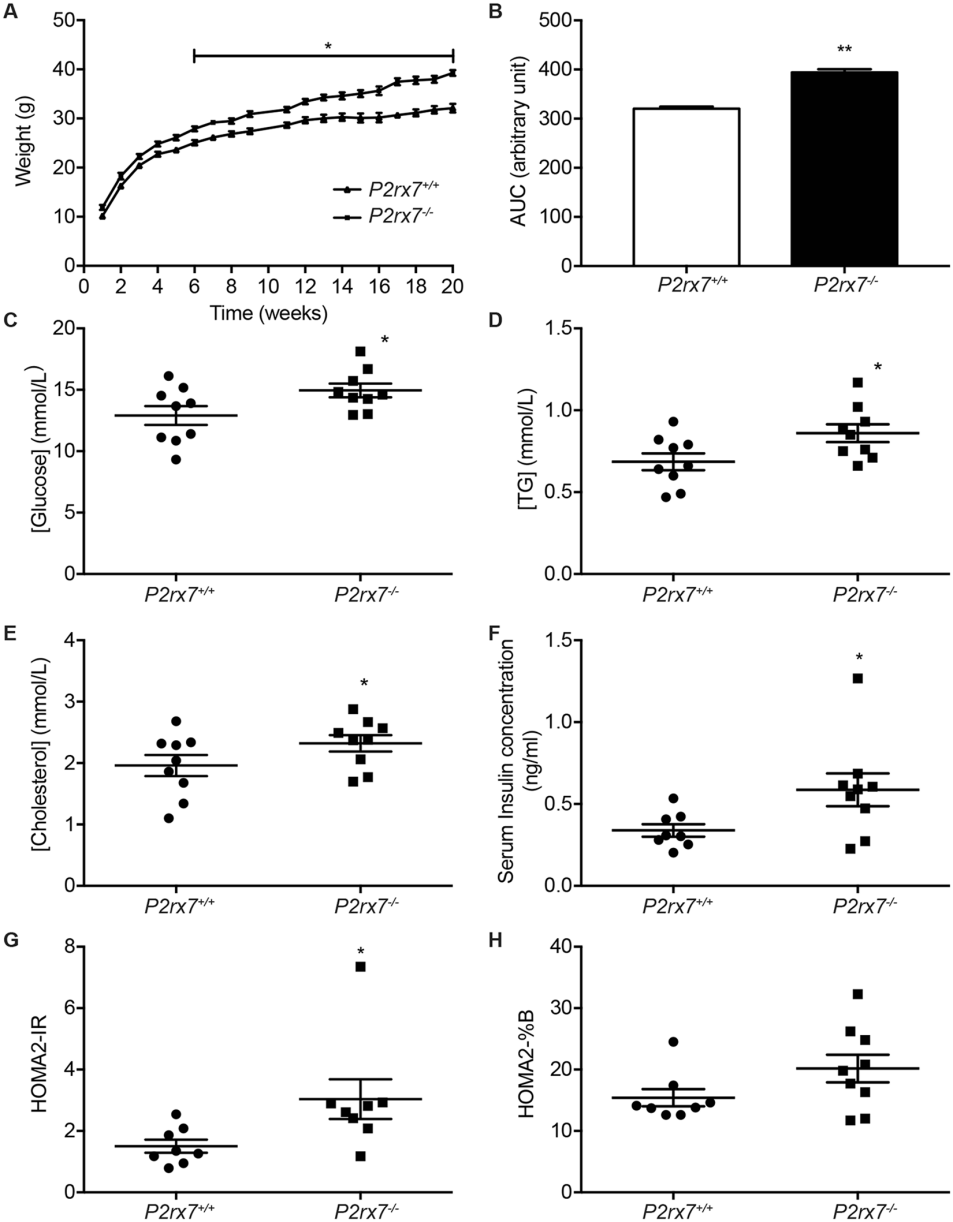
*P2rx7* ^−/−^ mice have increased body weight and abnormal glucose, insulin, triglyceride and cholesterol levels which could be associated to apparent resistance to insulin. (**A**) The weight of six *P2rx7* ^+/+^ and eight *P2rx7* ^−/−^ male mice was measured every week between 9–10 h AM starting at weaning (3-week-old mice) to 20-week-old mice. The results are expressed as the mean ± SEM and statistical significance was determined by an unpaired *t* test, where *p < *0*.*05*. (**B**) The Area Under the Curve (AUC) of the weight of experimental animals was determined by the trapezoidal method and expressed as the mean ± SEM. The statistical significance was determined by an unpaired *t* test, where **p < *0*.*01*. Serum biochemical analyses were realized on 12-week-old male mice (9 *P2rx7* ^+/+^ and 9 *P2rx7* ^−/−^) following 6h of diurnal fasting. Glucose (**C**), triglyceride (TG) (**D**) and total cholesterol (**E**) concentrations were determined using an automated clinical analyzer as described in the materials and methods section, whereas serum insulin concentration (**F**) was measured by an ELISA test. Results are presented as the mean ± SEM, where *p < *0*.*05* as determined by unpaired *t* tests. The HOMA2-IR (**G**) and HOMA2-%B (**H**) indices were calculated from the glucose and insulin levels found in panels C and F using the HOMA calculator presented in the M&M section. Results are expressed as the mean ± SEM. Statistical significance was determined by an unpaired *t* test, where *p < *0*.*05*.

In light of the [^18^F]-FDG PET and biodistribution assays as well as increase glycaemia in KO animals, it was not surprising to find a significant increase in the weight of *P2rx7* ^−/−^ mice as soon as 6 weeks of age when compared to *wild-type* animals (Fig. 4A). The weight increase was maintained through time as shown by the higher AUC value (Fig. 4B). Although this not the first report showing that *P2rx7* deficient mice have aberrant glycaemia and increase weight gain^13,14^, this study is the first to suggest that the increased glycaemia and weight gain might come from more efficient glucose uptake by the intestine. Furthermore, the weight increase measured in *P2X7* ^−/−^ animals was not the consequence of increase food uptake (supplemental Fig. S1A), a reduction of activity (supplemental Fig. S1B), nor a modulation of energy expenditure (supplemental Fig. S1C). The increase in plasma glucose concentration was validated in 12-week-old mice using an automated clinical analyzer (Fig. 4C), as previously described^27^. Increases in serum triglyceride (TG) (Fig. 4D) and cholesterol (Fig. 4E) levels were also measured in *P2rx7* ^−/−^ mice when compared to *P2rx7* ^+/+^ animals. The increase in blood glucose, TG and cholesterol concentrations was accompanied by a significant increase in serum insulin level in *P2rx7* ^−/−^ mice (Fig. 4F). The presence of high blood glucose concentration despite a significant increase in serum insulin level leads us to suspect that *P2rx7* ^−/−^ mouse might be insulin resistant. The HOMA2-IR test was used to determine the relative resistance of *P2rx7* ^−/−^ mice to insulin and pancreatic β-cell function (HOMA2-%B). As shown on Fig. 4G, *P2rx7* ^−/−^ animals had higher HOMA2-IR values, thus supporting the idea that knockout mice could be insulin resistant. However, the apparent insulin resistance does not appear to be a consequence of β-cells malfunction as HOMA2-%B values were not significantly different between the two animal groups (Fig. 4H).

### The absence of P2X7 expression increases the susceptibility of mice to glucose intolerance and insulin resistance

**Figure 5:**
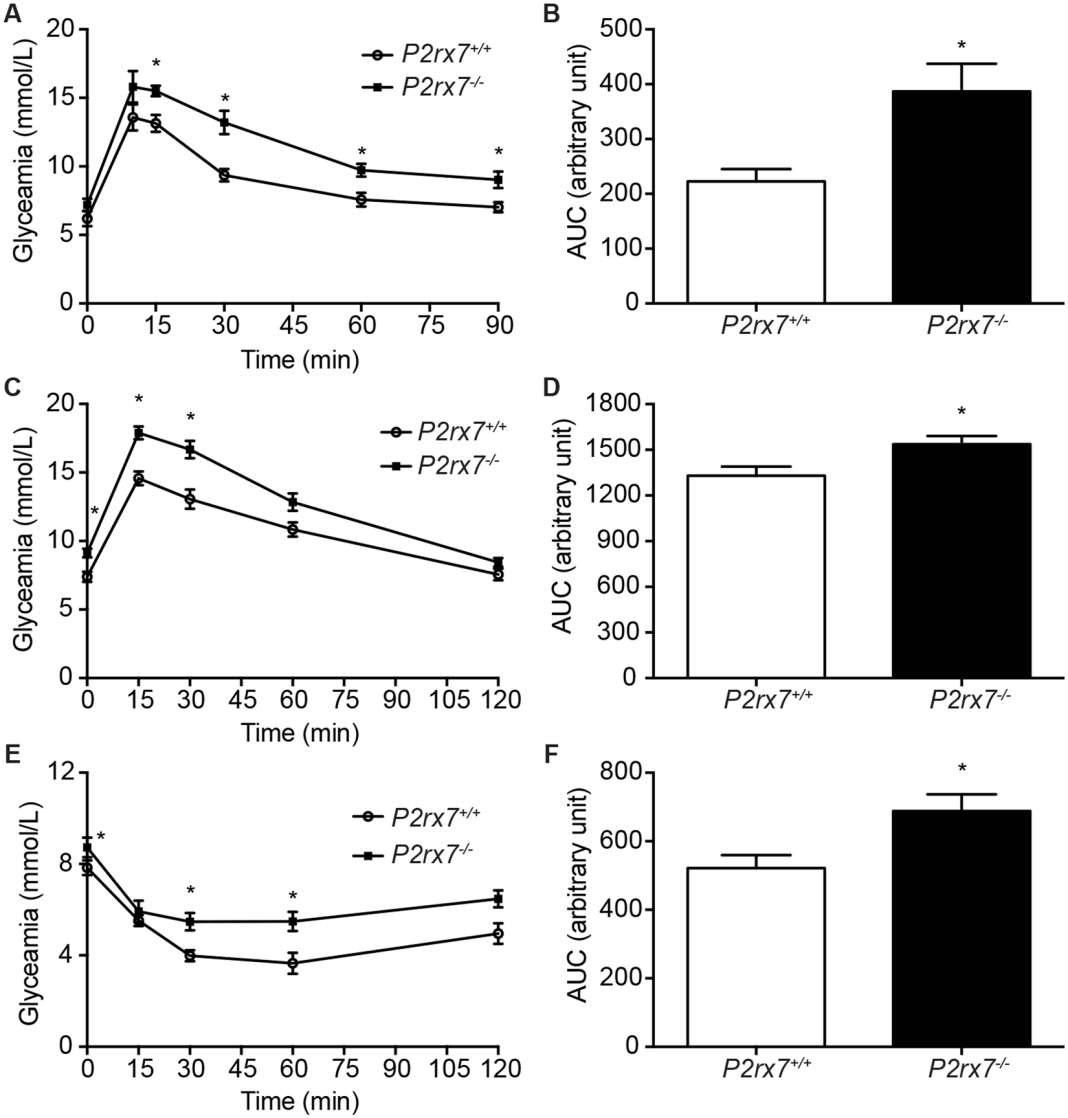
Glyceamia in *P2rx7* ^−/−^ mouse following glucose and insulin challenge. Blood glucose concentration during (**A**) OGTT, (**B**) IPGTT and (**C**) IPITT over time following the gavage of 50 mg of D-glucose or the intraperitoneal injection of 2 g D-glucose/kg or 0.5 U/kg of insulin following 6h of fasting. Results are presented as the mean ± SEM of 8 to 10 animals per group, where *p < *0*.*05* as determined by multiple *t* tests. (**B**,**D**,**F**) For each experimental condition, AUC was calculated using the trapezoidal method. Results are presented as the mean ± SEM of 8 to 10 animals per group, where *p < 0.05 as determine unpaired *t* tests.

In light of the HOMA2-IR test results, we further our investigation to determine if *P2rx7* ^−/−^ mice had impaired glucose tolerance and/or were insulin resistant. Oral glucose tolerance test (OGTT) results revealed that *P2rx7* ^−/−^ mice reached a significantly higher glycaemia 15 min following gavage (Fig. 5A). This difference in glycaemia was maintained up to 90 min post-gavage. The calculated AUC value, which is an indication of the blood glucose load, was significantly increased for the *P2rx7* ^−/−^ mice (Fig. 5B), suggesting that these mice could have a defect in their ability to manage glycaemia. To determine the ability of *P2rx7* ^−/−^ mice to clear glucose and manage insulin, animals were subjected to intraperitoneal glucose (IPGTT) and insulin (IPITT) challenges. The glycaemia was significantly higher in *P2rx7* ^−/−^ mice when compared to control littermates in both IPGTT (Fig. 5C,D) and IPITT (Fig. 5E,F) assays. Since the HOMA2-%B values were similar between the two groups, it seems that P2X7^−/−^ mouse intolerance to glucose could be linked to insulin resistance.

### *P2rx7*^−/−^ mice display hepatic steatosis

**Figure 6:**
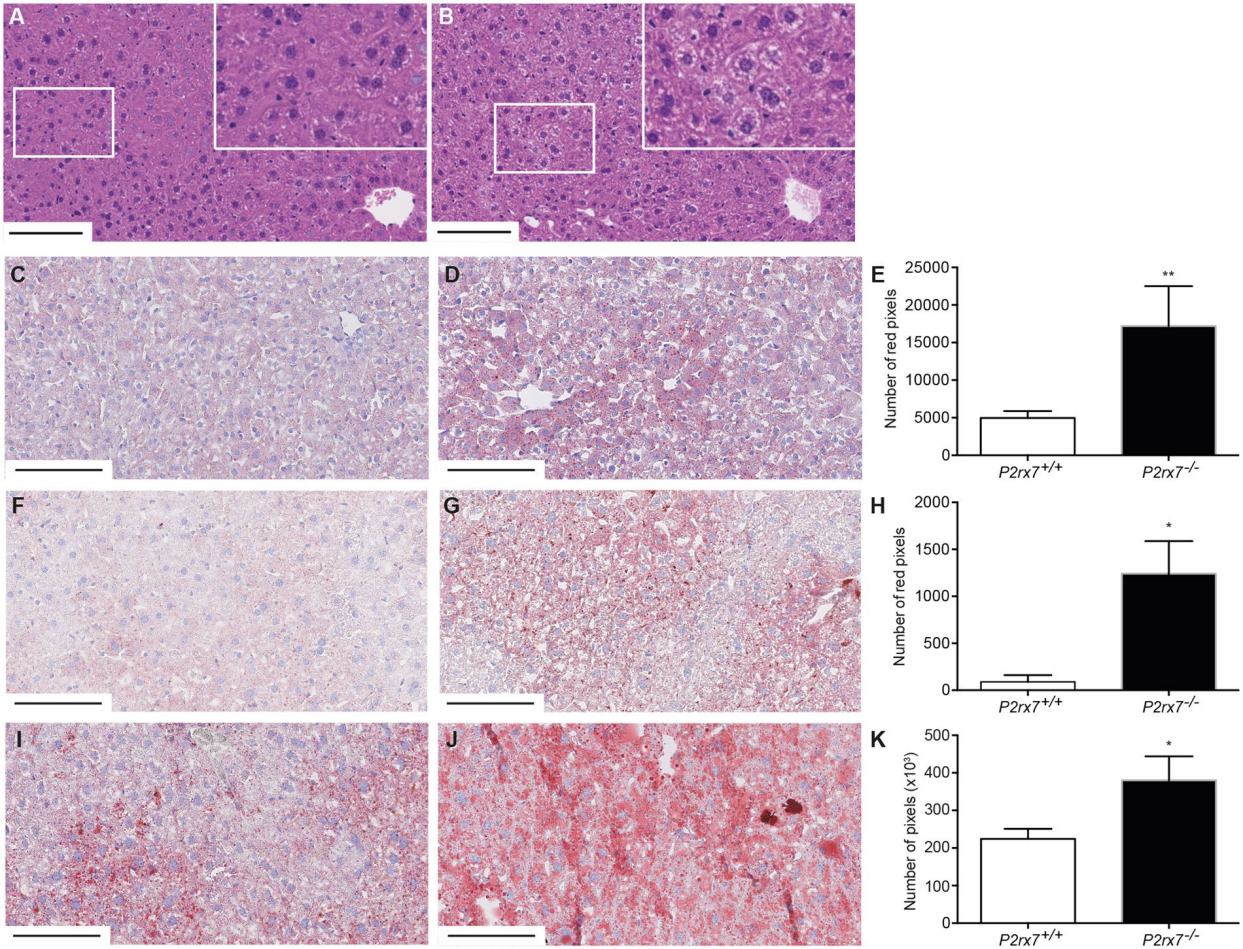
Oil red O staining reveals that *P2rx7* ^−/−^ mice accumulate lipids in their hepatocytes over time. Liver sections of 12-week-old (**A**) *P2rx7* ^+/+^ and (**B**) *P2rx7* ^−/−^ mice were H&E stained and lipid-enriched microvacuoles could be observed in the liver of knockout animals (inserts). Magnification of 40x where scale bars = 100 μm, and inserts are a zoom of the select zone showed by the white box. Oil red O staining for 21-day-old (**C–E**), 12-week-old (**F–H**) and 52-week-old (**I–K**) mice showed increase staining in *P2rx7* ^−/−^ animals (**D, G, J**) as compared to control littermates (**C**,**F**,**I**) over time. Magnification of 20x, where scale bars = 100 μm. Red-colored pixels were quantified as described in the M&M section and results presented as the mean ± SEM of three independent fields per mice for (**E**) four 21-day-old *P2rx7* ^+/+^ and 4 *P2rx7* ^−/−^ mice, (**H**) eight 12-week-old mice per group and (**K**) 7 *P2rx7* ^+/+^and 13 *P2rx7* ^−/−^ 52-week-old mice. Statistical significance was determined by comparing the pixel averages using an unpaired *t* test, where *p < *0*.*05* and **p < *0*.*01*.

Histologic analyses of H&E-stained liver sections of 12-week-old mice (Fig. 6A,B) revealed the presence of vacuoles, microvacuoles in particular, in the liver of *P2rx7* ^−/−^ animals (Fig. 6B) as compared to control mice (Fig. 6A). Oil red O staining of liver sections showed that lipid droplets accumulate in hepatocyte cytoplasmic vacuoles in *P2rx7* ^−/−^ mice as soon as 21 days postnatal (Fig. 6C–E; supplemental Fig. S2). The accumulation of hepatic lipid increases with times as shown by the mark staining in 3 months old (Fig. 6F–H) and 52-month-old (Fig. 6I–K) *P2rx7* knockout animals. The lipid deposition was mainly associated with macrovacuoles formation, with sparse microvacuoles also present. Although the vacuole distribution was mostly centrilobular, some lipid vacuoles were also observed in the midzonal region. Reddish-colored pixels were quantified as described in the materials and methods section and are presented in histograms (Fig. 6E,H,K). These results are confirming the accumulation of lipids in hepatocytes and the development of hepatic steatosis in *P2rx7* knockout mice when compared to age-matched control animals.

### Genes associated with liver lipid metabolism are downregulated in *P2rx7*^−/−^ mice

**Figure 7:**
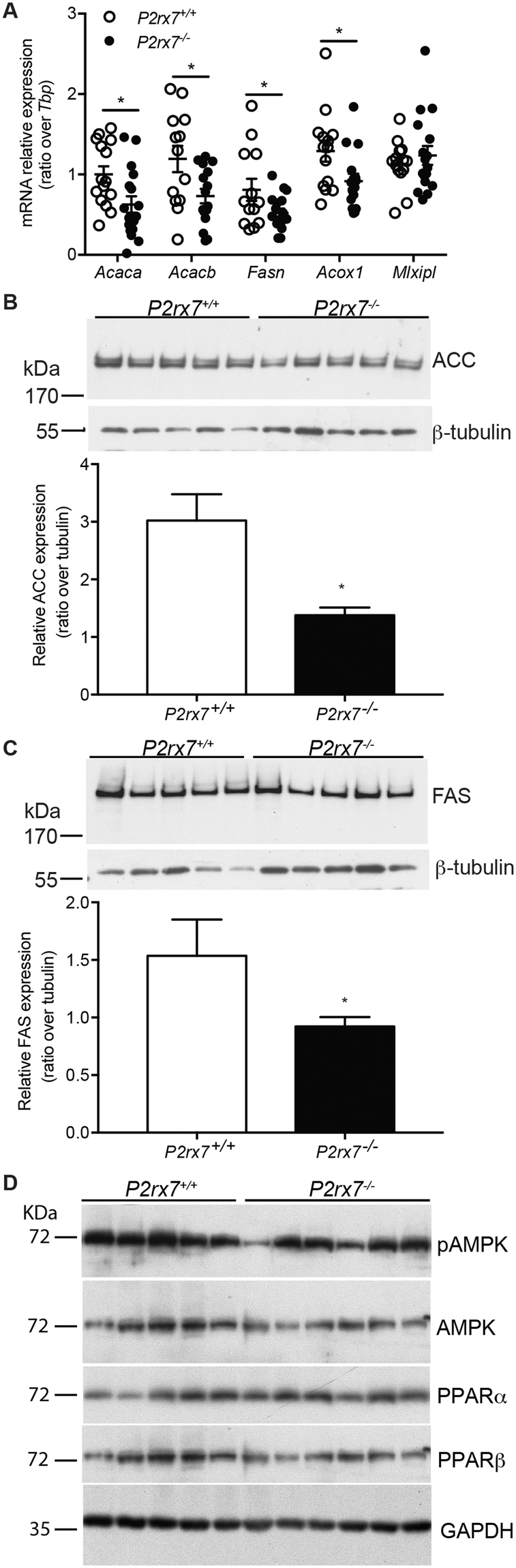
The expression of *Acaca*, *Fasn* and *Acox1* is downregulated in the liver of *P2rx7* ^−/−^ mice. (**A**) Livers of control wild-type littermates (open circles) and of *P2rx7* ^−/−^ mice (close circles) were harvested and mRNA extracted prior to quantitative real-time PCR assay. The relative mRNA expression was expressed as the ratio of the expression over *TATA-box binding protein* gene expression (*Tbp*). Results are presented as the mean ± SEM, where *p < *0*.*05* as determine by multiple *t* tests. Liver lysates were also obtained and (**B**) ACC and (**C**) FAS protein expression determined by Western blots. β-Tubulin expression was used as a control of protein integrity and to ensure equal loading. Signal intensity was quantified by densitometry and reported as the mean ± SEM of the ratio of protein expression over β-tubulin, where *p < *0*.*05* as determine by an unpaired *t* tests. (**D**) Hepatic levels of AMPK phosphorylation of Thr172 and protein expression for AMPK, PPARα, and PPARβ determined by Western blots. GAPDH expression was used as a control of protein integrity and to ensure equal loading.

Accumulation of lipids in hepatocytes might arise from increased lipid synthesis, or decreased lipid oxidation amongst other factors^28^. In this context, we analyzed the mRNA expression of acetyl-CoA carboxylase alpha and beta (*Acaca*, *Acacb*), fatty acid synthases (*Fasn*) that are involved in *de novo* synthesis of fatty acids, and acyl-CoA oxidase 1 (*Acox1*), which is key for lipid peroxisomal β-oxidation^29^. Despite the increased lipid accumulation in the liver of *P2rx7* ^−/−^ mice, we measured a significant reduction in *Acaca*, *Acacb*, *Fasn* and *Acox1* expression (Fig. 7A). The reduced gene expression of *Acaca*, *Acacb* and *FasN* was accompanied by a reduction of protein expression as well (Fig. 7B and C). Indeed, using a pan-ACC antibody we observed and quantified a significant reduction in the expression of ACC in the liver of *P2rx7* ^−/−^ mice as compared to control animals (Fig. 7B). Similarly, FAS protein expression was significantly reduced in the liver of knockout mice (Fig. 7C). On the opposite, no difference was observed for the expression of the *Mlxipl* gene that encodes for the carbohydrate-responsive element-binding protein (ChREBP) (Fig. 7A). AMPK, PPARα and PPARβ are well-described metabolic regulators and modulators of the above-described genes^30–33^. However, western blot analysis of AMPK, PPARα and PPARβ liver expression as well as AMPK phosphorylation of Thr172 did not revealed any significant difference between the two animal groups (Fig. 7D).

## Discussion

The ATP-gated P2X7 receptor regulates multiple physiological functions under normal and pathological conditions. Amongst the recent functions associated with this particular receptor, the regulation of energy metabolism has emerged^13,14,34,35^. Here, we showed that the loss of P2X7 receptor expression lead to the increase expression of GLUT2 at the apical compartment of *P2rx7* ^−/−^ mouse enterocytes under basal fasting condition. These results confirmed our previous finding that showed the internalization of GLUT2 following the stimulation of the P2X7 receptor in intestinal epithelial cell lines^4^. In fact, the apical localization of GLUT2 in *P2xr7* ^−/−^ mouse was similar to its expression in enterocytes of insulin resistant mouse^24^. The apical expression of GLUT2 was also observed in enterocytes of *ob*/*ob* and high fat-fed mice and in patients suffering from morbid obesity^36^. It was suggested that the apical localization of GLUT2 might favor glucose entries in the bloodstream and could thus contribute to the increase of glycaemia^36^. We could not find a significant overall difference in the efficiency of glucose absorption in the intestine using live PET imaging, which we believed was mainly due technical limitations as described above. However, the biodistribution analysis clearly showed a mark reduction of [^18^F]-FDG/glucose levels in the proximal intestinal region suggesting a rapid efficient absorption and transport of the glucose solution by the intestine of *P2rx7* ^−/−^ mice as compared to *P2rx7* ^+/+^ littermates. Few studies have used PET imaging to study live enteral glucose absorption. One of these studies has determined the distribution pattern of radiolabeled glucose derivative 60 min following gavage^37^. The reported distribution was similar to the pattern we measured in this work for *P2rx7* ^+/+^ animals. At this time, it was concluded that SGLT1 was the main cellular entity responsible for the absorption of glucose by enterocytes^37^. We cannot argue against the importance of SGLT1 in this process, however, our work also points out at an important contribution of GLUT2 in this process. We have shown that GLUT2 expression was increased at the apical compartment of *P2rx7* ^−/−^ enterocytes as well as at the basolateral side of these cells. We cannot rule out that the contribution of GLUT2 to the overall increase in blood glucose level might also come from the modulation of GLUT2 expression at the basal compartment of enterocytes thus favoring glucose exit toward the bloodstream. Nevertheless, the overall effect resulting from the absence of a functional P2X7 in enterocytes was an increase in enteral absorption of glucose and a significant augmentation in the level of circulating glucose. In fact, we have shown that [^18^F]-FDG rapidly accumulates in the highly vascularized muscle shoulder and blood of *P2rx7* ^−/−^ mice when compared to *P2rx7* ^+/+^ animals. Hence, the glycaemia in male *P2rx7* ^−/−^ mouse was increased in OGTT assays when compared to control *P2rx7* ^+/+^ littermates. The apparent discrepancy between the levels of circulating glucose and the [^18^F]-FDG activity measured in the heart of *P2rx7* ^−/−^ mouse could be explained by the diabetic phenotype and the presence of hepatic steatosis in *P2rx7* ^−/−^ mice. Indeed, it was showed that there is a drastic shift toward the utilization of fatty acids as the sole source of energy in the diabetic heart^26^. Hence, it was observed that diabetic patients with liver steatosis have decreased myocardial glucose uptake^38^.

The increase glycaemia that we measured in *P2rx7* ^−/−^ mice under basal fasting condition and following oral glucose challenges might also come from the reduced capacity of peripheral organs to adequately absorb glucose as we showed using IPGTT. We observed that the blood glucose concentration was higher 15 and 30 min following the glucose injection as compared to control animals. Similar results were obtained in *P2rx7* ^−/−^ mice fed a high-fat/high-glucose diet^13^. Glucose absorption by peripheral organ is mainly regulated by the presence of insulin and organs capacity to respond to circulating insulin. In this study, along with elevated glycaemia values, we measured elevated insulin concentrations in *P2rx7* ^−/−^ animals; thus suggesting that knockout animals could produce insulin but that this production was not sufficient to regulate glycaemia. The higher glycaemia measured in P2X7^−/−^ mice despite elevated serum insulin concentrations could be a sign of insulin resistance. Higher HOMA2-IR values in *P2rx7* ^−/−^ mice and significant higher glycemic values in intraperitoneal insulin tolerance tests in KO animals are supportive of this idea. Although euglycemic clamp tests could have better confirmed this finding, HOMA2-IR and HOMA2-%B values integrate normalized factors that give an accurate estimation of insulin resistance (IR) and β-cell functions (%B)^39–41^. In a previously published study, it was shown that the absence of P2X7 receptor expression reduced the overall pancreatic secretion in cell preparation isolated from mice^42^, and that *P2rx7* ^−/−^ mice had impaired β cells function^13^. However, in this study, the calculated HOMA2-%B values were similar between *P2rx7* ^+/+^ and *P2rx7* ^−/−^ mice, thus suggesting that pancreatic β-cell malfunction was not involved in terms of regulating the response to glucose challenges. In fact, the HOMA values that we obtained in our study are comparable to data that were measured in the fat-induced diet in the *ob*/*ob* mice model of insulin resistance^36^. These results are supporting the idea that the loss of P2X7 expression and function might also contribute to insulin resistance along with the increase efficiency of enterocytes to absorb and transport glucose.

In the liver, activation of the P2X7 receptor was associated to tissue injury by promoting fibrosis^43^, inflammation and oxidative stress^44^. Hence, peritumoral P2X7 expression in hepatocellular carcinoma was linked to an unfavorable prognostic for patient survival^45^. In our study, along with the apparent insulin resistance, high blood glucose levels and dyslipidemia, we observed an important accumulation of lipid droplets in hepatocytes of *P2rx7* ^−/−^ mice that is often associated with hepatic steatosis^46^. Hepatic steatosis is generally accompanied by the increase expression of *Acaca*, *Acacb*, *Fasn* and *Acox1* in the liver to compensate for the presence of free fatty acid^29^. Surprisingly, we measured a reduction in the expression of these genes in the liver of KO animals, as well as in the expression of ACC and FAS at the protein level. However, the reduction of *Acaca* and *Acacb* expression in the liver of *P2rx7* ^−/−^ mice could explain the presence of hepatic fat since the abrogation of *Acaca* and *Acacb* expression in double knockout mice leads to an accumulation of hepatic fat^47^. The reduction in Acox1 expression that we measured in our animal model was also observed in the *Acaca* and *Acacb* double knockout mouse model^47^. Similarly, the loss of *Acox1* expression was reported in a mouse model of high-fat diet-induced hepatic steatosis^48^. It was argued that the decrease β-oxidation resulting from the reduction of *Acox1* expression could drive the lipid processing pathway towards lipid synthesis and away from catabolism, thus to accumulation of hepatic lipids^48^. Moreover, the disruption of *Acox1* expression leads to the development of lipid microvesicules in steatosis liver^49^, similar to some of the observations we made in our study. Furthermore, it was reported that ACACA and FAS protein expression was significantly reduced in the liver of obese patients diagnosed with severe steatosis^28^. Given that signs of hepatic steatosis are already visible in 21-day-old mice, we could argue that our 3-month mice are suffering from advance liver steatosis as shown in Fig. 6G. This finding correlates well with the increased blood glucose levels; increased body weight, dyslipidemia and apparent insulin resistance found in the 3-month-old *P2rx7* ^−/−^ mice, as all of these parameters could be associated with liver steatosis^47^.

As an endocrine organ, the intestine secretes numerous gastrointestinal hormones regulating the metabolism^50,51^. This unique organ is also the center of nutrient absorption, including glucose. In this context, we previously reported that activation of the P2X7 by ATP lead to a reduction of glucose absorption and transport by IEC *in vitro* ^4^. In this study using *P2rx7* ^−/−^ mice, we validated these previous observations and established that P2X7 was an important factor to modulate intestinal glucose absorption and transport using live PET imaging. Furthermore, we have provided strong evidence that P2X7 might contribute to the establishment of a metabolic dysregulation, mainly with regards to glucose and lipids homeostasis. These are metabolic conditions that are increasingly prevalent worldwide. Hence, our results suggest that regulation of P2X7 activity through the use of antagonists as potential therapies for inflammatory diseases and cancer^52^ might be cautiously examined for potential metabolic adverse effect. Nonetheless, the use of P2X7 ligands to control enteral glucose transport offers an interesting perspective in the management of glycaemia. However, to develop this type of intervention we will have to develop strategies to target the P2X7 receptor expressed by IECs as previously reported^4^. As such, the systemic administration of P2X7 ligands might not be the solution as it could lead to immunoregulatory responses. In this context, orally available Pfizer CE-224,535 and GSK1482160 P2X7 receptor antagonists are potential candidates^53,54^. However, the action of both molecules was reported in the blood stream of patients, thus having systemic effect. Hence, the enteric distribution and activity of both compounds remain to be determined. Nevertheless, these studies clearly illustrate that targeting the P2X7 receptor in pathologies is far from being a fantasy. Furthermore, for this study we have developed an innovative modality of using [^18^F]-FDG and live PET imaging to follow enteral glucose transport. This particular modality in the use [^18^F]-FDG could lead to innovative approaches to study glucose metabolism and biodistribution in live organism such as human.

## Methods

### Animals

Adult *B6*.*129P2-P2rx7* ^*tm16ab*^/*J* and *C57BL*/*6J* mice were purchased from Jackson Laboratory (Bar Harbor, ME). *B6*.*129P2-P2rx7* ^*tm16ab*^/*J* mice were backcrossed with *C57BL*/*6J* mice for 10 generations. Experiments were performed with F10 and following generations. Mice genotype was determined using the previously described NaOH-based genomic DNA extraction protocol^55^. The genotype was determined by PCR amplification using the following oligonucleotide primers: 5′-TCACCACCTCCAAGCTCTTC-3′ for wild-type animals (*P2rx7* ^+/+^), 5′-GCCAGAGGCCACTTGTGTAG-3′ for *P2rx7* ^−/−^ mouse and a common reverse primer 5′-TATACTGCCCCTCGGTCTTG-3′. Mice were housed as previously described^56^. As indicated, prior to some experiments, mice were starved for 6 h during the daytime to circumvent the metabolic and catabolic stress induced by longer nocturnal starvation period as reported^57,58^.

### Monitoring mouse food uptake, locomotion and energy expenditure

The Promethion High-Definition Room Calorimetry System was used for the indirect calorimetry studies (GA3, Sable Systems, Las Vegas, NV). Prior to acquisition eight-12-week-old male mice (4 *P2rx7* ^+/+^ and 4 *P2rx7* ^−/−^) were acclimated to cages for 48 h. A standard 14 h light- 10 h dark cycle was maintained and animals had access *ad libitium* to standard chow and water. For the experiments, mice had *ad libitium* access to standard chow and water. Data acquisition and instrument control were coordinated by MetaScreen v. 1.6.2 and the obtained raw data was processed using Expe-Data v. 1.4.3 (Sable Systems, Las Vegas, NV) using an analysis script detailing all aspects of data transformation. For this study, we focused on food uptake, animal locomotion and energy expenditure (EE). Results for food uptake (gram), locomotion (meter) and EE (kcal/hr) were presented as the mean ± SEM in function of the circadian cycle.

### Tissues processing

Excised jejunums were flushed of debris using PBS as previously described^56^, and either used for epithelial cell enrichment for RNA and protein expression analysis or fixed in Carnoy solution (60% methanol, 30% chloroform, 10% glacial acetic acid) and used for paraffin embedding for immunohistochemical analyses. Liver sections were also isolated and used for RNA and protein expression analysis or fixed in 3% PFA and embedded in paraffin for subsequent histologic studies. Alternatively, liver sections were placed in a 30% saccharose cryoprotective solution as described by Drover^59^ prior to OCT inclusion and Oil Red O staining by the Electron Microscopy and Histology Research Core from the Université de Sherbrooke Faculty of Medicine and Health Sciences. For the Oil Red O staining quantification, image acquisition from stained liver sections was performed using a Hamamatsu Nanozoomer apparatus under bright field illumination. The zoom was set at 20x and the presence of red pixels measured using Adobe Photoshop and a predefine array of different red shades enriched for magenta color. An average of at least 3 different fields per mice were measured for the quantification analysis.

### GLUT2 immunohistochemistry

Male *P2rx7* ^+/+^ and *P2rx7* ^−/−^ mice were starved for 6 h prior to experiments. Jejunum sections (5 μm) were deparaffinized, rehydrated and treated with Dako peroxidase blocking solution (Agilent Technologies Canada, Inc., Mississauga, ON). Jejunum slides were treated with the avidin/biotin blocking kit (Vector laboratories, Brockville, ON) and non-immunogenic sites blocked with PBS containing 0.1% Tween 20 and 2% BSA (PBT-BSA). Slides were incubated with goat polyclonal anti-GLUT2 (C-19) (Santa Cruz Biotech, Santa-Cruz, CA) antibodies at a dilution of 1:100 in PBS-BSA for 2 h at room temperature (RT). Slides were washed in PBS and incubated with biotinylated rabbit anti-goat IgG (1:200; Vector laboratories) in PBT-BSA for 1 h at RT followed by incubation with the Vectastain biotinylated horseradish Peroxydase H Elite ABC Kit (Vector laboratories) and revealed with the Dako DAB and chromogen solution (Agilent Technologies Canada, Inc.). Slides were counterstained with a hematoxylin solution following the manufacturer instructions (Sigma-Aldrich, Oakville, ON) and mounted using VectaMount mounting medium (Vector laboratories). Slides were scanned at 40 X with Hamamatsu Nanozoomer 2.0-RS system and analysed with the NDP scan software.

### Live glucose absorption by Positron Emission Tomography (PET) and [^18^F]-FDG biodistribution assays

PET imaging and ^18^F-FDG biodistribution were assessed on three-month-old male mice starved for 6 h prior to the experiment as described above. Mice were gavaged with 100 μl of a 500 mg/mL glucose solution containing 3 to 5 MBq of ^18^F-FDG using a 24G × 1” W/1.25 mm gavage needle (Cadence Inc., Staunton, VA). Animals received the radioactive glucose solution in less than 15 seconds, rapidly anesthetized in 2–4 minutes by isoflurane inhalation in a pre-saturated chamber and placed in a LabPET/Triumph PET/CT scanner (Gamma Medica, Northridge, CA) for a 90 min live acquisition of ^18^F-FDG distributions. Dynamic PET acquisition data were reconstructed as a series of 26 images frames (10 × 1 minute, 16 × 5 minutes) and radioactivity uptake in selected organs was extracted by region-of-interest analysis. Measured radioactivity data, corrected for ^18^F decay (half-life of 110 min) referred to time of injection, were converted as the % of injected dose/g of tissue (%ID/g) given that 1 g of tissue is estimated at 1000 μl following virtual tissue reconstruction. PET image visualization, analysis and 3D rendering were performed using the AMIDE software^60^. Following the 90 min acquisition, organs of interest, as presented in Fig. 2, were harvested to measure the ^18^F-FDG biodistribution. Briefly, whole tissues were placed in 12 × 75 mm tubes and ^18^F-FDG emissions measured using a Packard Cobra II E5003 gamma counter (Perkin Elmer, Waltham, MA). The %ID/g values were corrected based on the initial injected dose corrected for the ^18^F-FDG-decay factor.

### Western immunoblotting

Intestinal epithelial cells were isolated from the isolated jejunums using the BD Cell recovery solution as previously described^56^, and homogenized in Triton buffer (40 mM Tris pH 7.5, 150 mM NaCl, 1 mM EDTA, 1% Triton X-100, 0.2 mM sodium orthovanadate, 50 mM sodium fluoride, 40 mM β-glycerophosphate, 0.1 mM phenylmethylsulfonyl fluoride and protease inhibitor mixture from Sigma-Aldrich). Livers were also harvested and homogenized as described above. Protein samples were processed as described^61^, with the omission of the heat-denaturation step. Proteins were separated on a 7% SDS-PAGE and transferred to polyvinylidene fluoride membranes for protein immunoblotting. Immunoblotting for protein expression was performed using a 1:750 dilution of rabbit polyclonal anti-P2X7 (APR-004) antibody (Alomone Labs, Jerusalem, Israel), 1:1000 dilution of rabbit polyclonal anti-GLUT2 (H67) antibody, 1:1000 dilution of goat anti-PPARα and anti-PPARβ antibodies (Santa Cruz Biotech, Santa Cruz, CA), 1:1000 dilution of rabbit polyclonal anti-AMPK and rabbit monoclonal anti-phospho AMPK (Thr172) (New England Biolabs, Ltd., Whitby, ON, Canada), 1:2000 dilution of rabbit polyclonal anti-ACC and of rabbit monoclonal anti-FAS antibodies (New England Biolabs, Ltd., Whitby, ON, Canada). The antibodies were diluted in PBS containing 5% BSA or in PBS containing 0.1% Tween-20 and 0.5% fish gelatin and incubated overnight at 4 °C. Specific protein bands were detected using a 1:10000 dilution of horseradish peroxidase (HRP)-conjugated anti-rabbit IgG (GE Healthcare Life Sciences, Mississauga, ON) or 1:5000 HRP-conjugated donkey anti-goat IgG (Santa Cruz Biotech, Santa Cruz, CA) and visualized on autoradiographic film using the Millipore chemiluminescence system. Signal normalization was performed as described previously using a 1:1000 dilution of mouse monoclonal anti-Vinculin or anti-GAPDH (Millipore, Etobicoke, ON), or 1:5000 dilution of rabbit monoclonal anti-β-Tubulin antibody (New England Biolabs, Ltd., Whitby, ON, Canada) and 1:10000 dilution of HRP-conjugated anti-mouse or anti-rabbit IgG (GE Healthcare Life Sciences). Crude lung homogenates, isolated from *P2rx7* ^+/+^ animals, was used as a positive control for P2X7 expression.

### Serum glucose, triglycerides (TG), cholesterol and insulin quantification

Blood was collected by cardiac puncture of the right ventricle of 6 h-fasted mice. After 30 min at room temperature, samples were centrifuged at 3,000 × g for 15 min at 4 °C. Serum insulin levels were quantified by ELISA using the rat/mouse insulin Millipore ELISA kit (EZRMI-13K). Serum glucose, cholesterol and TG concentrations were measured using commercially available kits (Siemens Healthcare Diagnostics, Deerfield, IL) on a clinical analyzer (Dimension XPand Plus, Dade Behring Inc.) as described^27,62^. Relative insulin resistance and pancreatic β-cell function were estimated using the Homeostasis Model Assessment 2-Insulin Resistance test (HOMA2-IR) and HOMA2-%B (http://www.dtu.ox.ac.uk/homacalculator/index.php).

### Glucose and insulin tolerance tests

Oral glucose tolerance tests (OGTT), intraperitoneal glucose tolerance tests (IPGTT) and intraperitoneal insulin tolerance tests (IPITT) were realized on 3-month-old male mice starved for 6 h as described above. For OGTT, mice were gavaged with 100μl of a 500 mg/ml D-glucose solution using a 24 × 1” W/1.25 mm gavage needle (Cadence Inc.). For IPGTT and IPITT, 2 g D-glucose/kg or 0.5 U/kg of insulin (Novolin^®^ge Toronto, Novo Nordisk Canada, Inc., Mississauga, ON) were respectively injected intraperitoneally. Blood glucose values were determined from whole venous blood obtained from mice tails using a glucose monitor (FreeStyle Lite; Abbott Diabetes Care). Results were plotted as the variation of glycaemia over time using GraphPad Prism and the area under the curve (AUC) calculated by the trapezoidal methods to establish the overall amount of blood present in the circulation after 90 min.

### Quantitative real-time PCR analysis

Total RNA was isolated from the liver of 12-week-old mice using the ToTALLY RNA Total RNA Isolation Kit (Thermo Fisher Scientific) and RNA purified Qiagen RNeasy minikit (Qiagen, Toronto, ON). Complementary DNA was synthesized from 2 μg of purified RNA as previously described^63^. Quantitative real-time PCR analyses were realized as previously reported^63^ using the mouse sequence-specific primers for *Acaca*: 5′-GATGAACCATCTCCGTTGCG-3′ and 5′-GAGCCAATTATGAATCG GGACTG-3′, *Acacb*: 5′-TTCCCCAGCCAGCAGATAGC-3′ and 5′-CTTCATGTAGCCACGGGTCC-3′, *Fasn*: 5′-GATGAAGAGGGACCATAAAGAATAA-3′ and 5′-GCACTTGATGTGAG GGGAGAT3′, *Acox1*: 5′-CCGCCACCTTCAATCCAGAG-3′ and 5′-CAAGTTCTCGATTTCTCGACGG-3′, and *Mlxipl*: 5′-CTGGGGACCTAAACAGGAGC-3′ and 55′-GAAGCCACCCTATAGCTCCC-3′. Gene expression was normalized to *Tbp* gene expression as previously reported^64^.

### Statistics

Results are expressed as the mean ± standard error of the mean (SEM). Statistical significance was determined by a multiple comparison unpaired *t* test for data having a normal Gaussian distribution or corrected for Mann-Whitney for non-normal distributed results. The number of replicates and animals for each experiment are presented in figure legends.

### Study approval

All procedures were performed according to the protocol #217-13BR that was approved by the Université de Sherbrooke Animal Care Committee and the Canadian Guidelines for Care and Use of Experimental Animals.

### Availability of data and material

All data generated or analyzed during this study are included in this article and its supplementary information files.

## Electronic supplementary material

Live transition of the [^18^F]-FDG/glucose solution in the intestine of mice.

Supplemental Figures
