# Supplementary material for: The loss of P2X7 receptor expression leads to increase intestinal glucose transit and hepatic steatosis
Source: Sci Rep. 2017 Oct 10;7:12917. doi: 10.1038/s41598-017-13300-8 (PMC5635021; doi:10.1038/s41598-017-13300-8)
Supplement: Supplementary file 2 — Supplemental Figures [file 41598_2017_13300_MOESM2_ESM.pdf]

The loss of P2X7 receptor expression leads to increase intestinal glucose transit and hepatic steatosis

Guillaume Arguin¶, Jean-François Bourzac¶, Morgane Placet, Caroline M. Molle, Michel Paquette, Jean-François Beaudoin, Jacques A. Rousseau, Roger Lecomte, Mélanie Plourde, and Fernand-Pierre Gendron

¶ Both authors have contributed equally to this work.

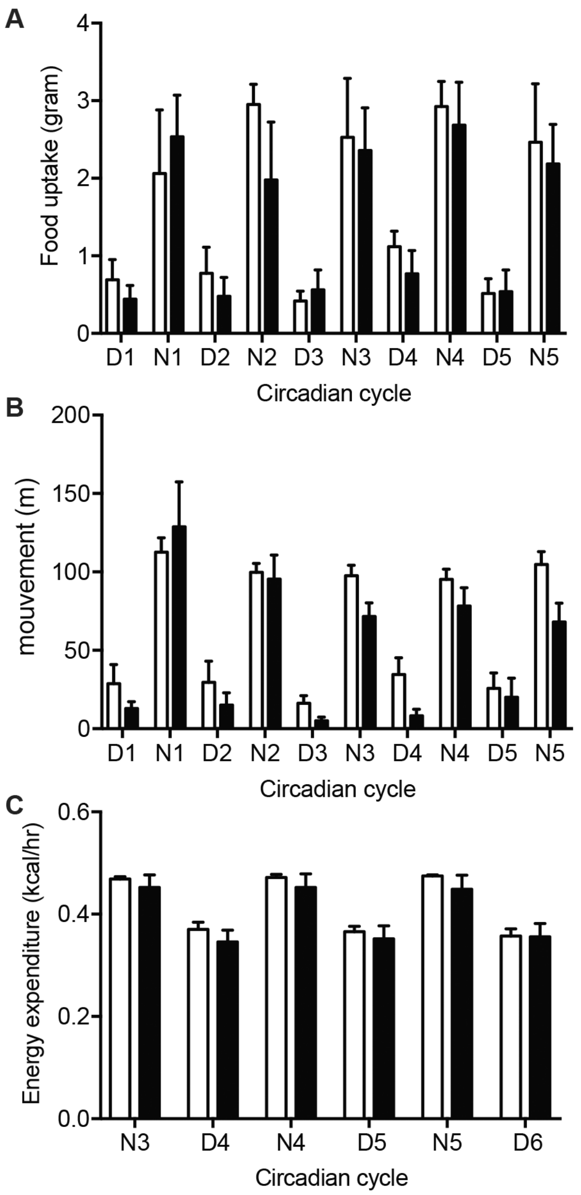

Supplemental Fig. 1. The increase weight observed for *P2rx7*$^{-/-}$ mice was not the result of increase food uptake or a reduction of animal movement or energy expenditure. The Promethion high-definition room calorimetric system was used to automatically monitored (**A**) food uptake, (**B**) animal movement and (**C**) total energy expenditure (EE). Four *P2rx7*$^{+/+}$ and four *P2rx7*$^{-/-}$ mice were used for this study. Results are presented as the mean ± SEM over a 5-day period for food uptake and movement and over a 3-day period for EE, where D = day time (6h – 20h) and N = night time (20h – 6h). No significant difference could be measured between both animal groups.

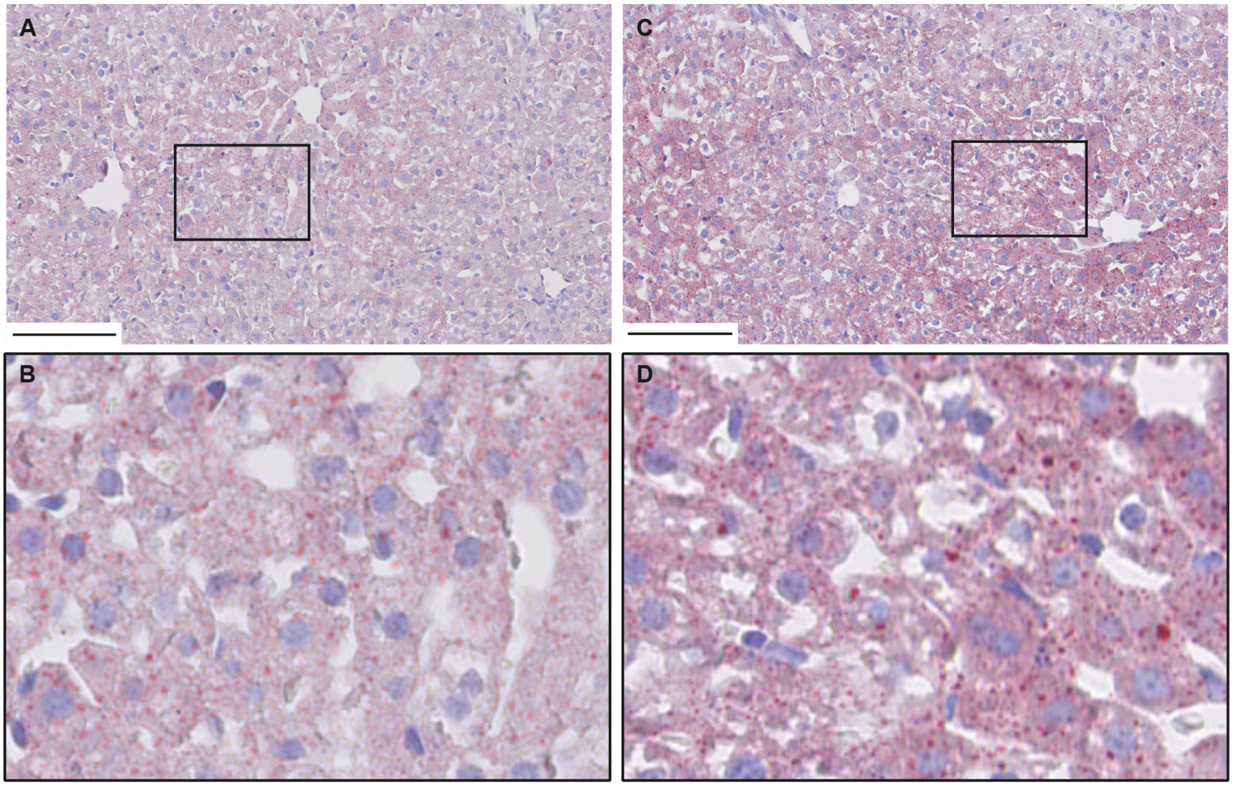

Supplemental Fig. 2. Increase magnification of Oil red O-stained hepatic tissue sections of 21-day old mice showing cytoplasmic lipid deposistion. A) Oil red O-staining at 40x for *P2rx7*$^{+/+}$ mouse and (**B**) Higher zoom of the zone delineate by the black box in panal A showing weak lipid staining in hepatocyte cytoplasm. **C**) Lipid staining with Oil red O at 40x magnification for *P2x7*$^{-/-}$ animals, with a higher magnification of the delineated zone (black box) shown in panel **D**. Scale bars = 100 μm.
